# Simple Preparation of Tetrazole Chitosan Derivatives Which Exhibit High Catalytic and Antibacterial Activity

**DOI:** 10.3390/polym17121657

**Published:** 2025-06-14

**Authors:** Anton R. Egorov, Linh V. Nguyen, Nkumbu D. Sikaona, Omar M. Khubiev, Roman A. Golubev, Abel M. Maharramov, Rovshan H. Nazarov, Alexander G. Tskhovrebov, Vasili V. Rubanik, Vasili V. Rubanik, Aleh V. Kurliuk, Anatoly A. Kirichuk, Wanjun Liu, Andreii S. Kritchenkov

**Affiliations:** 1 Department of Human Ecology and Bioelementology, RUDN University, 6 Miklukho-Maklaya St, 117198 Moscow, Russia; linhno2109@gmail.com (L.V.N.); nkumbusikaona534@gmail.com (N.D.S.); ihubievomar1@gmail.com (O.M.K.); asdfdss.asdasf@yandex.ru (R.A.G.); alexander.tskhovrebov@gmail.com (A.G.T.); kirichuk-aa@rudn.ru (A.A.K.); 2 Institute of Technical Acoustics NAS of Belarus, Ludnikova Prosp. 13, 210009 Vitebsk, Belarus; v.v.rubanik@tut.by (V.V.R.); iakustika@mail.ru (V.V.R.J.); 3 Organic Chemistry Department, Baku State University, 23 Z. Khalilov Street, AZ1148 Baku, Azerbaijan; amaharramov@bsu.edu.az; 4 Institute of Chemistry of Additives, Beyukshor Highway, Block 2062, AZ1029 Baku, Azerbaijan; rovsenhafiz@mail.ru; 5 Department of General and Clinical Pharmacology, Vitebsk State Medical University, Frunze av. 27, 210009 Vitebsk, Belarus; aleh.kurliuk@gmail.com; 6 Shanghai Frontiers Science Center of Advanced Textiles, College of Textiles, Donghua University, Shanghai 201620, China

**Keywords:** chitosan, electrochemistry, hydrazone–azide coupling, aldol reaction catalyst, antibacterial activity

## Abstract

Chitosan is a natural, biocompatible, biodegradable, and non-toxic polymer that has consistently garnered the attention of researchers in the development of new materials across various applications. Typically, to impart the desired properties to chitosan, chemical modification is necessary. Therefore, the development of simple and convenient methods for the chemical modification of chitosan is crucial in polymer chemistry. In this work, the approaches of Click chemistry and the necessary electrochemistry, which have recently illuminated the chemistry of chitosan, were combined to achieve a straightforward and efficient synthesis of new tetrazole chitosan derivatives. This was accomplished through electrochemical coupling. The proposed synthesis method is simple, convenient, and fast, hence allowing for the easy production of low- (10%), moderate- (30%), and highly substituted (65%) tetrazole chitosan derivatives. The highly substituted chitosan derivatives exhibit high activity as catalysts for the aldol reaction, achieving almost 100% conversion in just 15 min. Notably, these derivatives enable the aldol reaction to be catalyzed in water, aligning with one of the key principles of green chemistry. Furthermore, the new tetrazole chitosan derivatives demonstrate significant *in vivo* antibacterial effects in the treatment of peritonitis in rats. The primary mechanism of their antibacterial action is the disruption of the bacterial cell membrane integrity.

## 1. Introduction

Polymer science has been significantly enriched over the past few decades, thanks to the ingenious approach of Click chemistry [1], pioneered by Nobel Prize Laureate Barry Sharpless, along with his colleagues and followers. This breakthrough has revolutionized organic synthesis [2,3]. The Click polymerization technique [4] is synthetically convenient for polymer-analogous transformations, mild reaction conditions, and high yields, among the advantages that specifically highlight the excellence of Click chemistry in polymer science [5,6].

The Click reaction methodology has also significantly enriched chitosan chemistry [7]. Previously, this field was constrained by the traditional organic reactions and further complicated by chitosan’s solubility, which is often limited to acidic aqueous media [8]. The introduction of Click reactions into chitosan chemistry has led to the development of new, highly effective chitosan derivatives with elegant chemical structures. These derivatives are utilized in targeted drug delivery, cell culture matrices, wound healing, cosmetics, food processing, packaging materials, sorbents, catalysts, and more [9]. However, most of the publications in the Click chitosan chemistry domain primarily focus on the alkyne–azide cycloaddition reaction, which produces *tri*azole chitosan derivatives [10,11,12].

However, related tetrazole chitosan derivatives are considerably less frequently encountered in chitosan chemistry [13]. In conventional organic chemistry, tetrazoles are well-established and extensively studied [14]. Tetrazoles, obtained through classical organic synthesis, have found wide application, primarily in chemistry, as effective organocatalysts for several important organic transformations [15,16,17], and in the biomedicine field, where many tetrazoles exhibit significant antimicrobial activity [18,19,20]. It stands to reason that various tetrazole chitosan derivatives would be of interest, both as catalysts and as antimicrobial compounds, since they could combine the beneficial properties of chitosan with the tetrazole heterocyclic system. Nonetheless, the number of tetrazole chitosan derivatives described and studied remains relatively minimal [13,21]. This is likely because the existing methods for their synthesis are labor-intensive, have low yields, and are often accompanied by side reactions, as detailed in a recent review [13].

This gap can be addressed through the development of new approaches for the preparation of tetrazole chitosan species that avoid these disadvantages. Many of the electrochemical Click reactions fall into this category [22], and they have the potential to revitalize chitosan chemistry. For example, the direct arylation technique has recently been successfully applied in chitosan chemistry [23], and the electrochemical synthesis of rare benzothiazole chitosan derivatives has also been achieved [24]. Given the general interest in the electrochemistry of this group, we have focused on the recently described effectual electrochemical hydrazone–azide dipolar cycloaddition reaction [25]. This reaction between organic azides and arylhydrazones requires low-current (~10 mA) lithium chlorate as a supporting electrolyte, and it takes place in an undivided electrochemical cell on an RVC anode and a Pt cathode. This reaction proceeds extremely quickly under mild conditions in virtually quantitative yields, thereby aligning with the concept of a Click reaction. Despite its promise, this attractive reaction has never been used in polymer chemistry. Therefore, to explore this reaction, a decision was made to establish a new, simple, and convenient route to produce tetrazole chitosan derivatives, which are of primary interest, both as catalysts and as antibacterial agents.

## 2. Materials and Methods

Chitosan (15, 60, and 160 kDa; degree of deacetylation, 80%), *p*-chlorohydrazine, 4-formylbenzoic acid, 1-ethyl-3-(3-dimethylaminopropyl)carbodiimide (EDC), *N*-hydroxysuccinimide (NHS), Trimethylsilyl Azide (TMSN_3_), acetonitrile, acetone, and 4-nitrobenzaldehyde were purchased from Sigma-Aldrich (St. Louis, MO, USA). Other chemicals and solvents were obtained from commercial sources and used as received. The reticulated vitreous carbon anode was furnished from a commercial reticulated vitreous carbon (RVC) sheet, and the platinum cathode was from BVB-Alians (Moscow, Russia).

The ^1^H NMR spectra were recorded on a Bruker AVANCE spectrometer (Bruker, Karlsruhe, Germany) operating at a frequency of 400 MHz. The samples were dissolved in D_2_O with 1% (wt.) CF_3_COOH.

The apparent hydrodynamic diameter and ζ-potential of nanoparticles in water were estimated at room temperature (~20 °C) by the DLS method using a Photocor Compact-Z instrument (Photocor, Moscow, Russia) at λ = 659 nm and θ = 90°.

SEM images were obtained by using an electron microscope JEOL JSM-6490LV (JEOL, Tokyo, Japan) at 15 kV and a SEM detector with an electron beam size of 30, in high vacuum. The samples were coated with a layer of Pt in a JEOL auto fine coater JFC-1600 (40 s at 40 mA, resulting in a nominal film thickness of 20 nm) to improve their conductivity.

The viscosity of the chitosan solutions in 0.3 M NaCl/2% acetic acid was measured at 20 °C in an Ubbelohde viscometer. The intrinsic viscosity [η] of the chitosan was calculated by the extrapolation of the dependence ln(η_r_) × C^−1^ to an infinite dilution by using the least squares method. The viscosity average molecular weight (MW) of the chitosan was calculated by using the Mark–Kuhn–Houwink–Sakurada equation: [η] = 3.41 × 10^–3^ × MW^1.02^ [26].

SEM images were obtained by using the JEOL JSM-5410LV (JEOL, Tokyo, Japan) scanning electron microscope, equipped with an Integrated Calibration and Application Tool (INCA) system and an Energy-Dispersive X-Ray (EDS) microanalysis detector, operated at 20 kV.

IR spectra were measured on a Shimadzu IRPrestige-21 spectrometer (Shimadzu, Kyoto, Japan).

**HCs** species: Chitosan (1 g) was dissolved in 1% acetic acid (50 mL). Then, 4-((2-(*p*-chloro)hydarzinylidene)methyl)benzoic acid (0.15, 0.6, or 1.2 equivs.), ethylene dichloride (EDC) (0.15, 0.6, or 1.2 equivs.), and N-hydroxysuccinimide esters (NHS) (0.15, 0.6, or 1.2 equivs.) were added. The reaction mixture was stirred (at 150 rpm) for 2 h at room temperature.

**TCs** species: A 10 mL three-necked round-bottomed flask was charged with a solution of hydrazone chitosan derivative (**HCs**, 0.3 mmol) in a solvent system, which consisted of 4 mL of MeCN and 2 mL of MeOH. The dissolution of **HCs** is described in detail above. Thereafter, the reaction flask was charged with lithium perchlorate (LiClO_4_) (0.3 mmol) and TMSN_3_ (1.5 mmol). The flask was equipped with a condenser, a reticulated vitreous carbon (RVC) anode (100 PPI, 1 cm per 1 cm per 1 cm), and a platinum plate (1 cm per 1 cm) cathode. The reaction mixture was electrolyzed at a constant current (10 mA) at 0 °C for 2 h (2.5 F) under constant stirring (150 rpm). The reaction was quenched by the addition of acetone (4 mL). The precipitated polymers were washed with acetone, dissolved in water, dialyzed against water, and freeze-dried.

For the catalytic experiments, 4-nitrobenzaldehyde (100 mg) and the catalyst (5 mg) under testing were dispersed in 15 mL of a water/acetone 6/1 *v*/*v* mixture and stirred at room temperature for 5, 10, 15, 30, and 60 min. Then, the reaction was quenched by the addition of 45 mL of acetone, and the precipitated catalyst was removed and separated by centrifugation (8000× *g*). The solvent was evaporated under vacuum to give solid residues, which were quantitatively analyzed by ^1^H NMR spectroscopy to evaluate the conversion of the starting aldehyde into the aldol condensation product.

The *in vitro* antibacterial activity of the chitosan-based polymers prepared was evaluated as described elsewhere [27]; the MTT test was performed according to its conventional procedure [24,28]; the *in vivo* antibacterial effect and *in vivo* toxicity were completely estimated, in accordance with a previously published procedure [24]. Briefly, the *in vitro* antibacterial effect of the tested samples against *S. aureus* (RCMB 010027) and *E. coli* (RCMB 010051) was estimated by using the agar-well method. The agar, cooled to 45 °C, was poured into sterile Petri dishes. The agar solidified, and after that, 0.2 mL of the suspension of the tested bacteria with a titer of 10^6^ CFU/mL was applied to its surface. Thereafter, the Petri dishes were left for between 1 and 2 h to absorb the inoculum. After that, wells with a diameter of 6 mm were made on the surface of the agar by using a sterile drill. Then, the wells were filled with 0.2 mL of a solution of the sample under test at a concentration of 0.1%. After that, the Petri dishes were placed in a thermostat and kept at 30 °C for 48 h. The antibacterial activity was determined by measuring the diameter of the inhibition zone (in mm). Each inhibition zone was measured three times by using a caliper to obtain an average value. Ampicillin and gentamicin were used as the antibacterial standard drugs.

To estimate the *in vitro* cell viability (the *in vitro* toxicity) 3-(4,5-dimethylthiazol-2-yl)-2,5-diphenyltetrazolium bromide (MTT) test was conducted. The solutions of the tested samples were prepared by serial dilutions in an α-Minimum Essential Medium (alpha-MEM) culture medium. A 0.1 mL volume of each solution was added to a confluent monolayer of cells, cultured in a 96-well plate. HEK293 cells were incubated for 24 h at 37 °C in an atmosphere containing 5% CO_2_. The cells were washed twice with phosphate-buffered saline (PBS), and then, 0.1 mL of 3-(4,5-dimethylthiazolyl)-2,5-diphenyltetrazolium bromide (MTT; 0.5 μg/mL) in PBS was added and incubated for 4 h. The supernatant was then replaced with 0.1 mL of 96% ethanol, and the absorbance was measured at a wavelength of 535 nm.

Male Wistar rats of a 3-month-old line weighing between 180 and 200 g were used in the work for the evaluation of the *in vivo* antibacterial activity. The animals were kept in a vivarium under the conditions of a 24 h photo regime, a controlled temperature (22 ± 2 °C), air humidity of 65 ± 10%, and free access to water and standard feed. The experiments were carried out in the first half of the day (10:00–13:00 Moscow time) in compliance with the rules for the humane treatment of laboratory animals. During the implementation of this work, the general requirements of the European Convention Directive 2010/63/EU of 22 September 2010 were met. The middle third of the right half of the abdominal wall was shaved for the white male Wistar rats, after which the skin was treated with an alcoholic solution of iodine. A microbial mixture was used as an infectious agent, and the cavity in physiological saline was injected with 3 mL of a polymicrobial suspension consisting of the same amount of *S. aureus* and *E. coli* strains. After a total of 31 h following infection in the control groups, 200 μL of exudate was collected with a sterile syringe. In the experimental groups, rats were injected with a solution (0.8 mg per 300 µL) of the chitosan-based system, chitosan in the form of hydrochloride, ampicillin, or gentamicin, after 24 h. After a total of 7 h after treatment, 200 µL of exudate was taken. Each exudate obtained was diluted in physiological saline for an hour, and 6 10-fold dilutions of 100 μL were prepared, which were applied evenly on a Petri dish with meat-peptone agar. Colonies were counted 24 h after incubation in a thermostat at 37 °C. Subsequently, the colony-forming units (CFUs) were recalculated per 1 mL of exudate.

The *in vivo* toxicity experiments were carried out, also in the male Wistar rats of a 3-month-old line weighing between 180 and 200 g, in the first half of the day (10:00–13:00 Moscow time), in compliance with the rules for the humane treatment of laboratory animals mentioned above. The animals were divided into experimental and control groups. For the experimental rat group, the middle third of the right half of the abdominal wall was shaved, the skin was treated with an alcoholic solution of iodine, and the rats were injected once with a solution (0.8 mg per 300 µL) of the tested sample. From the moment of injection and over the course of 14 days, the behavior patterns of the animals (typical eating and drinking habits, unimpaired coordination, standard breathing rates and depths, and regular bowel movements) were assessed. The frequency of urination and the color of the urine were also assessed.

The statistical significance of the differences between the samples was determined by a one-way analysis of variance (ANOVA). Upon performing the ANOVA, the differences between the sample means were determined by using Tukey’s post hoc test. This was conducted at a significance level of *p* < 0.05 by using the JMP 7 software (SAS Campus Drive; Cary, NC, USA).

## 3. Results and Discussion

### 3.1. Chemistry

#### 3.1.1. Electrochemical Preparation and Characterization of Tetrazole Chitosan Derivatives

**Scheme 1:**
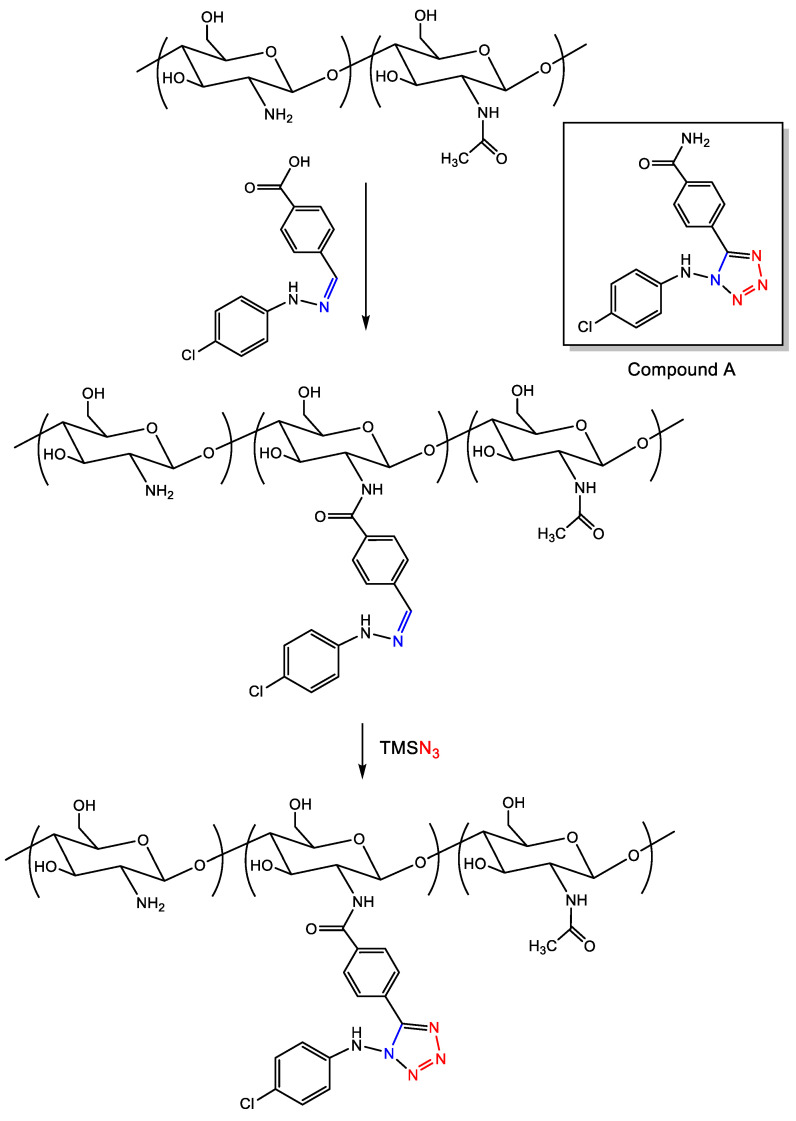
Electrochemical preparation of tetrazole chitosan derivatives.

For the electrochemical preparation of tetrazole chitosan derivatives, chitosan of low (15 kDa), medium (60 kDa), and high (160 kDa) molecular weight was employed. The transformation of chitosan into its tetrazole derivatives, proposed in this work, is a consequence of two simple stages, i.e., the preparation of hydrazone chitosan derivatives (Scheme 1, **HCs**, **route A**) followed by the electrochemical coupling of the resultant hydrazones with azide (Scheme 1, **TCs**, **route B**).

The first stage includes the treatment of chitosan with hydrazone, named the 4-((2-(*p*-chloro)hydarzinylidene)methyl)benzoic acid-bearing carboxylic group. The hydrazone is readily available from commercial *p*-chlorohydrazine and 4-formylbenzoic acid by a conventional *Ad_N_-E* reaction. The conjugation of chitosan with hydrazone (Scheme 1, **HCs**, **route A**) was achieved by the classical carbodiimide method, which is widely used in chitosan chemistry to form an amide bond between the primary amino group of chitosan and the carboxyl functionality of a suitable reactant [29]. The required degree of substitution in the resulting polymers can be easily adjusted by simply increasing the reagent/chitosan ratio. Thus, hydrazone chitosan derivatives with low (0.10), moderate (0.30), and high (0.65) degrees of substitution, starting from chitosan of low, medium, and high molecular weight, were prepared (series **HCs**, see Table 1).

**Figure 1:**
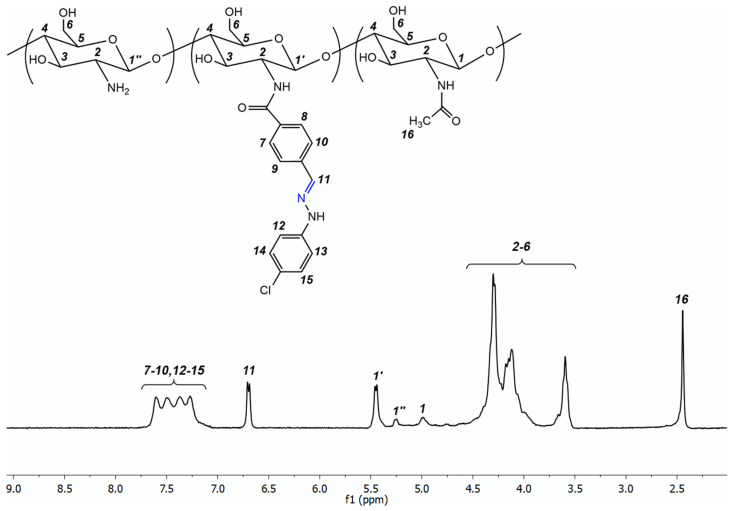
^1^H NMR spectrum of **HCs-0.65-L**.

The chemical structure of the resultant polymers was confirmed by ^1^H NMR spectroscopy. The typical spectrum is shown in Figure 1. The degree of substitution (DS) of the resultant polymers was calculated according to DS = I(***11***)/I(***1*** + ***1′*** + ***1″***), where I is the integral intensity of the corresponding protons (see Figure 1).

Since the next stage of the synthesis (electrochemical coupling, Scheme 1, **route B**) requires non-aqueous solvents (solvent system MeCN/MeOH 2/1 *v/v*), the solubility of the resultant polymers’ **HCs** in organic solvents was not of interest. The polymers with a high degree of substitution (**HCs-0.65** series) are soluble in polar organic solvents, such as DMSO, DMF, MeOH, EtOH, and most importantly in MeCN/MeOH 2/1 *v/v*. The moderately substituted polymers (**HCs-0.30** series) can also be dissolved in the MeCN/MeOH 2/1 *v*/*v* solvent system, but there is a certain procedure to follow. The polymer should be primarily swollen in MeCN and then dissolved by five freeze–thaw cycles. The subsequent, gradual drop-wise addition of MeOH, with vigorous stirring, allows the polymer to remain dissolved. This solution should be immediately used in further synthetic operations, since within 24 h, it turns first into a gel-like and then into a glassy mass. The situation is interesting in the case of low-substituted polymers (**HCs-0.10** series). In this case, the dissolution, only for the low-molecular-weight chitosan derivative **HCs-0.10-L,** was achieved. The dissolution of **HCs-0.10-L** was made possible by using MeCN with lithium perchlorate, followed by three freeze–thaw cycles. Methanol should then be added slowly, under stirring. In this case, it is essential that both MeCN and MeOH are anhydrous.

It is believed that the highly substituted polymers (**HCs-0.65** series) are capable of dissolving in organic solvents, since (*i*) they bear many non-polar aryl side substituents, and (*ii*) due to steric reasons, they prevent the cross-linking of the polymer chains by hydrogen bonds (which, for example, are characteristic of starting chitosan). By considering the moderately substituted polymers (**HCs-0.30** series), additional action of the freeze–thaw cycles is required for the final destruction of the system of interchain hydrogen bonds [30]. Finally, the blocking of the possibility of cross-linking macromolecules via hydrogen binding in the low-substituted chitosan derivative **HCs-0.10-L**, in addition to freeze–thaw cycles, also requires the addition of lithium salts. A similar approach by using lithium salts is very popular for dissolving chitin in dimethylacetamide [31].

**Figure 2:**
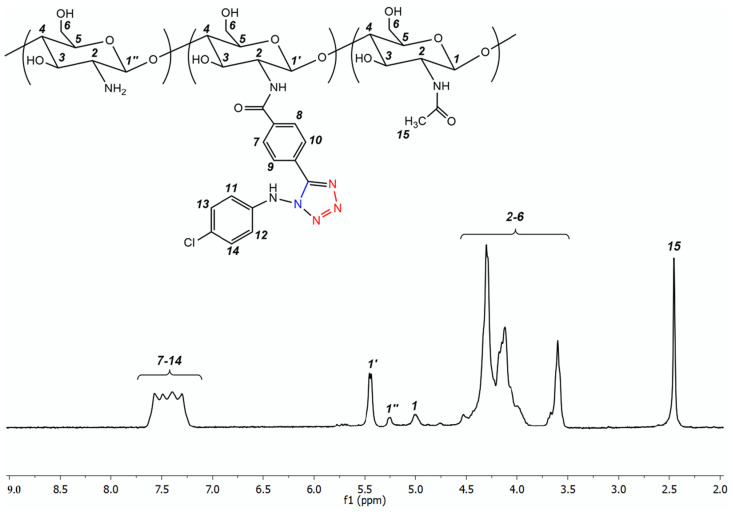
^1^H NMR spectrum of **TCs-0.65-L**.

The polymers of the **HCs** series, capable of dissolving in MeCN/MeOH 2/1 *v*/*v,* were involved in further electrochemical coupling with azide to furnish new tetrazole chitosan derivatives **(**Scheme 1, **TCs**, **route B**). The electrochemical interaction of **HCs** with TMSN_3_ proceeds smoothly at a constant current of 10 mA for 2 hours at 0 °C. Thus, the conditions described for the electrochemical [2+3] cycloaddition in so-called small-molecule chemistry [25] are suitable for chitosan chemistry, and these conditions allow for the rapid and convenient preparation of tetrazole chitosan derivatives. The complete conversion of **HCs** polymer series into the corresponding **TCs** species is reliably confirmed by using the NMR ^1^H spectroscopy (complete disappearance of the proton ***11*** signal, shown in Figure 1). The typical spectrum of the **TCs** polymers is shown in Figure 2.

The polymers that were prepared are readily soluble in water, but they lose their ability to dissolve in the organic solvents listed above, in comparison with the corresponding **HCs** species. This is apparently due to the introduction of a hydrophilic polar tetrazole ring to the side substituents of the macromolecules.

Particularly interesting in this regard are the low-substituted derivatives of low-molecular-weight chitosan **HCs-0.10-L**. These polymers are capable of self-assembling into nanoparticles that can form an aqueous nano-suspension, which is stable for at least 30 days. In addition, the mentioned nanoparticles are easily redispersed in water, and they can completely restore their starting hydrodynamic diameter and zeta potential value. The resulting self-assembled nanoparticles were characterized by using dynamic light scattering. The nanoparticles were characterized by a unimodal size distribution, and they have a hydrodynamic diameter that is quite rare for chitosan, i.e., about 30 nm. The nanoparticles exhibit a high positive zeta potential (about +48 mV). The high positive zeta potential is apparently due to the large number of amino groups that are available for protonation, and it is the high zeta potential that is the most important factor in the stability of nanoparticles in a suspension and their ability to redisperse [32,33].

**Figure 3:**
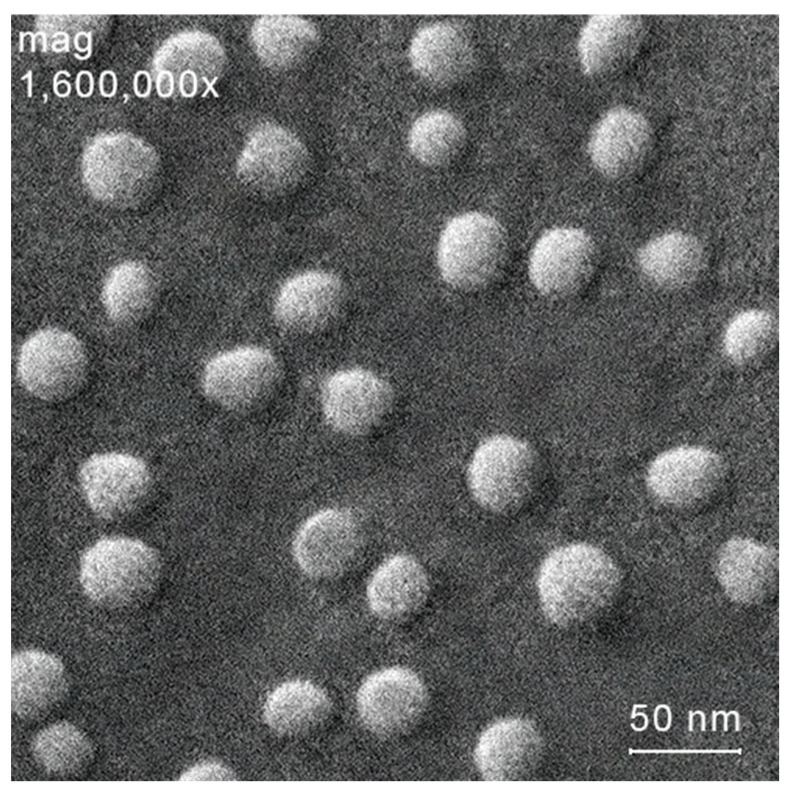
SEM image of self-assembled nanoparticles **HCs-0.10-L**.

The monodispersity of the resulting self-assembled nanoparticles and their sizes were also confirmed by using scanning electron microscopy (SEM). The SEM image of the **HCs-0.10-L** nanoparticles is shown in Figure 3, with the nanoparticles exhibiting a spherical shape. The spherical shape of the nanoparticles is of interest for their biological testing since spherical nanoparticles are generally considered the least toxic to mammalian and human cells [34].

#### 3.1.2. Catalytic Activity of Tetrazole Chitosan Derivatives

The aldol reaction, an important transformation in organic chemistry, often results in new carbon–carbon bond formation [35]. This reaction involves the interaction between two carbonyl compounds, one of which acts as a C-nucleophile (methylene moiety, due to a deprotonated alpha CH acid reaction center), and the other as a C-electrophile (carbonyl moiety, due to a carbonyl group) [36]. The aldol reaction is widely used in chemical synthesis, including large-scale processes (the industrial preparation of 1-butanol, 2-ethylhexanol, and pentaerythritol) and for several different monomers for the synthesis of high-molecular compounds [37]. In addition, the aldol reaction is of paramount importance in medicinal and bio-organic chemistry, especially in the synthetic preparation of physiologically active compounds (hapalosin, cryptophycin B, tetrahydrolipstatin, amphidinolide T1 macrolides, and many others [38]).

In traditional procedures, aldol condensation requires harsh conditions (strong bases and a high temperature) and is frequently complicated by several adverse reactions. These circumstances encourage researchers to search for highly efficient catalysts for the aldol reaction, especially the environmentally friendly types. In this context, both chitosan derivatives and various tetrazole-based compounds are attractive, and this has been demonstrated in recent works [39,40,41]. Inspired by these findings, the catalytic activity of the resulting tetrazole chitosan derivatives was tested in the current work.

**Scheme 2:**
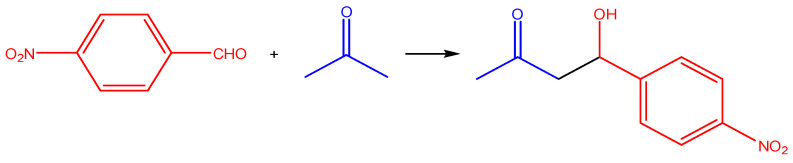
The model aldol reaction.

The model aldol reaction is shown in Scheme 2, and it involves acetone (C-nucleophile) and 4-nitrobenzaldehyde (C-electrophile).

To select the reaction medium, a recent publication in which Quignard et al. [42] carried out an aldol condensation process between cyclohexanone as a methylene component (C-nucleophile) and aliphatic or aromatic aldehydes as a carbonyl component (C-electrophile) in the most environmentally friendly solvent, i.e., water, was made reference to. The authors used chitosan aerogel as an organocatalyst. In the current work, a water/acetone 6/1 *v*/*v* mixture was employed because (*i*) acetone is a reactant in the model reaction (Scheme 2) and (*ii*) the solvent system dissolves the tested polymers as well as the reactants. Moreover, when the reaction is carried out in water/acetone 6/1 *v*/*v*, it can be quenched at any desired time by the simple addition of acetone. The large excess of the acetone added led to the precipitation of the catalyst (chitosan derivative) and allowed its easy removal by filtration from the reaction mixture. In this work, the reaction was quenched at 5, 10, 15, 30, and 60 min, while the catalyst and solvent were removed, and the solids were then analyzed by using ^1^H NMR spectroscopy to evaluate the product yield. The results of the experiment are summarized in Table 2.

Under the studied conditions (room temperature, solvent system water/acetone 6/1 *v*/*v*), the starting chitosans were unable to catalyze the model aldol reaction, even at 60 min after the start of the experiment, and no evidence was found of the formation of aldol condensation product. This is believed to be due to the absolute insolubility of chitosan in water at neutral pH values and the poorly developed surface of the chitosan catalyst. The low-substituted (**TCs-0.10-L**) and the moderately substituted (**TCs-0.30-L**, **TCs-0.30-M**, **TCs-0.30-H**) tetrazole derivatives of chitosan can catalyze a model aldol condensation reaction. The **TCs-0.10-Ls** are poor catalysts and they provide only ~26% conversion of the 4-nitrobenzaldehyde to the desired reaction product in 60 min. Conversely, in the **TCs-0.30** species, the reaction proceeded selectively and led to the complete conversion of the starting aromatic aldehyde into the corresponding aldol within 60 min. The best catalysts for the aldol reaction are the highly substituted polymers (**TCs-0.65-L**, **TCs-0.65-M**, **TCs-0.65-H**), which led to a selective 100% yield of the target product in just 15 min.

Thus, the catalytic effect of the electrochemically prepared polymers depended dramatically on their degree of substitution. This pronounced dependence led to the belief that the catalytic activity of the tetrazole derivatives of chitosan was due to the side substituent introduced into the polymer backbone of chitosan. To test the current hypothesis, compound **A** (Scheme 1) was prepared, and its high catalytic activity was determined in a model reaction. Notably, 15 min after the start of the reaction, it was found that the yield was also quantitative. However, a preparative separation of the aldol reaction product from catalyst **A** required column chromatography. The advantage of using polymers **TCs-0.65-L**, **TCs-0.65-M**, and **TCs-0.65-H** is that they not only exhibit high catalytic activity but can also be easily separated from the reaction mixture by simply adding excess acetone, followed by filtration. In addition, it was found that the polymer catalysts prepared did not lose their activity when reused at least 20 times.

### 3.2. Biological Activity

#### 3.2.1. In Vitro Antibacterial Activity Assessment

The tetrazole chitosan derivatives prepared are of clear interest as antimicrobial agents. Many tetrazole derivatives are used in biomedicine as antimicrobial, pharmacologically active compounds [43,44]. In addition, some tetrazole derivatives of chitosan were found to have exhibited good *in vitro* antibacterial activity, coupled with desirably low toxicity [21]. These exciting findings have inspired the testing of the electrochemically synthesized chitosan tetrazole species as antimicrobial agents. The first stage of this large work was focused on the *in vitro* primary assessment of the antibacterial effect to identify the so-called “leading systems”.

The agar-well diffusion method has proven to be a reliable tool for the *in vitro* testing of the antibacterial effect. This method is based on the ability of the molecules of antibacterial compounds to diffuse in agar media and form zones of inhibition in which the microorganisms under test, sensitive to the tested antibacterial agent, do not develop. Thus, given the same concentration of the test substance, a larger zone of inhibition of bacterial growth corresponds to a greater antibacterial effect.

Table 3 shows the *in vitro* assessment of the antibacterial activity of the prepared tetrazole-containing polymers in comparison with the starting chitosans and the commercial antibiotics, viz, ampicillin and gentamicin, against the model microorganisms, *Staphylococcus aureus* (Gram-positive) and *Escherichia coli* (Gram-negative).

The lowest antibacterial activity is a characteristic of the starting chitosan: its antibacterial effect is more than two times less than that of the conventional antibiotics, i.e., ampicillin and gentamicin. In addition, it was found that antibacterial activity in the studied range of the molecular weights (15, 60, and 160 kDa) was more pronounced for the low-molecular-weight chitosan (15 kDa). It is quite possible that this is the case since the low-molecular-weight chitosans are able not only to interact with the bacterial cell membrane, thus provoking its dysfunction with subsequent cell death, but also to penetrate the cell and bind the DNA polyanion [45,46]. The binding of chitosan to the DNA blocked its replication and transcription processes, and this inevitably led to the death of the microbial cell.

The antibacterial effect of the moderately substituted polymers and highly substituted derivatives is almost the same. This may be explained by the so-called “carpet mechanism” of action of the antibacterial agent [47]. According to the so-called “carpet hypothesis” about the mechanism of interaction between an antibiotic and a bacterial cell, the antibiotic molecules are located parallel to the surface of the cell membrane, thereby covering it, like a carpet. This led to the disruption of the self-organization of the lipid layer and destruction, hence, “biting off” of a section of the membrane. Binding to the bacterial cell in the case of the tetrazole derivatives of chitosan can be ensured, on the one hand, by the hydrophobic interactions, due to the introduced substituent and, on the other hand, by the protonated free amino groups (which can effectively interact with the anionic sites on the surface of the bacterial cell) [48].

It was assumed that the antibacterial effect of the moderately and highly substituted polymers obtained was primarily due to the substituent that was introduced into the chitosan polymer backbone. This hypothesis was confirmed by assessing the antibacterial effect of compound **A**. Compound **A** (Scheme 1) corresponds to the substituent introduced into the polymer chain, which exhibited an antibacterial effect comparable to moderately and highly substituted chitosan derivatives (even slightly more, and this was not surprising).

The most active polymers turned out to be low-substituted polymers **TCs-0.10-L**, which are capable of self-assembly into the corresponding nanoparticles. However, this goes against the previous general patterns, but in this case, a reasonable explanation for the observed fact is therefore offered. The literature data often indicate that polymer-based nanoparticles are characterized by a more pronounced antibacterial effect than the polymers in their native form [49,50]. In addition, the **TCs-0.10-L** nanoparticles are characterized by a high positive zeta potential value, which can ensure their very effective interaction with the anionic surface of the bacterium, hence disrupting the integrity of the membrane, the operation of ionic sediments, provoking the leakage of the contents of the bacterial cell into the external environment and the obvious and inevitable death of the bacterium [49].

#### 3.2.2. Permeabilization of Bacterial Cell Membranes

A reasonable hypothesis has been proposed to explain the antibacterial effect of chitosan and the various chitosan-based systems. It describes chitosan as a polycation that attacks the negatively charged bacterial cell membrane [51]. This leads, primarily, to the disruption of the ion pumps (due to their thin and vulnerable structural organization), thus provoking an osmotic imbalance that exposes the cell to its rupture [52,53]. Cell rupture, in turn, is accompanied by the leakage of intracellular contents (nucleic acids, proteins, etc.) into the external environment. The rate and intensity of leakage can serve as a measure of damage to the bacterial cell membrane. Thus, the tested chitosan-based system, which causes a more rapid and intense leakage of intracellular contents from a suspension of bacterial cells, is characterized by the greatest antibacterial effect due to a direct damaging effect on the bacterial membrane. The leakage of the intracellular contents from a bacterial suspension is accompanied by a sharp increase in optical density at 260 nm, and this made it convenient to monitor the leakage by using conventional spectrophotometry [54].

**Figure 4:**
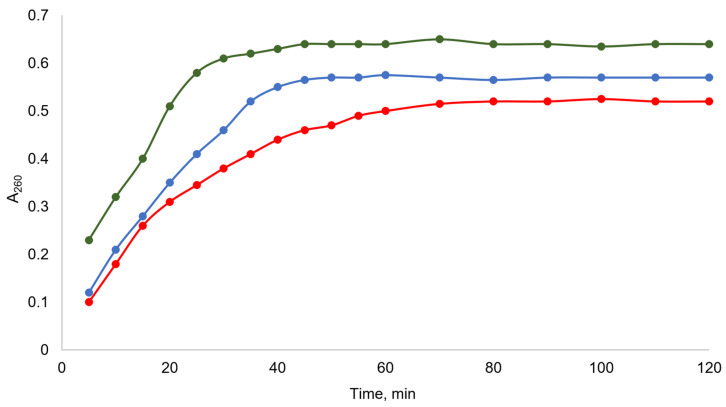
Release of bacterial cell contents absorbing at 260 nm from *E. coli* treated by chitosan (red), **TCs-0.10-L** (blue), or **TCs-0.30-L** (green). The relative standard deviation for all points is 5–10%.

In this work, the ability to damage the bacterial cell membrane (permeabilization of the bacterial membrane) for the leading chitosan-based systems identified in paragraph ***3.2.1*** (**TCs-0.10-L** and **TCs-0.30-L,** since they showed the maximum *in vitro* antibacterial effect) was assessed. In addition, their ability to damage the bacterial cell membrane with that of the starting chitosan was compared with a viscosity average molecular weight of 15 kDa. The results of this inornate but informative experiment are presented in Figure 4.

Gram-negative bacteria *E. coli* were used as model microorganisms. Gram-negative bacteria, unlike Gram-positive bacteria, have two membranes, between which there is a cell wall and a periplasmic space. This structure of the Gram-negative bacteria allows them to be more resistant to unfavorable conditions and also causes high resistance to cell rupture [55]. Thus, if the tested antibacterial agent causes the cell rupture of a Gram-negative microorganism, then it will definitely provoke the cell rupture of a Gram-positive bacteria.

Figure 4 shows that the least leakage of the contents of the bacterial cell was caused by the starting chitosan, and the greatest leakage was observed under the influence of the polymer **TCs-0.10-L**, which was prone to self-organization into the corresponding nanoparticles. This is evidenced by the A_260_ values on the plateau, which increased markedly, in the series of the chitosan (A_260_ = 0.520)—**TCs-0.30-L** (A_260_ = 0.573)—**TCs-0.10-L** (A_260_ = 0.641). In addition, the higher rate of development of the maximum leakage effect (the value corresponding to the time of reaching the plateau) also increased in the series chitosan (t_plateau_ = 70 min)—**TCs-0.30-L** (t_plateau_ = 50 min)—**TCs-0.10-L** (t_plateau_ = 40 min). These observations are entirely consistent with those observed in the initial *in vitro* evaluation of the antibacterial activity (paragraph ***3.2.1***). Thus, the results obtained from the *in vitro* experiments, with the model bacteria, allow for the conclusion that (*i*) the most active antibacterial systems among the synthesized chitosan derivatives are **TCs-0.10-L** or **TCs-0.30-L**, with **TCs-0.10-L** being the undoubted leader and (*ii*) the tested samples causing significant damage to the bacterial cell membranes and the cell walls, which appeared to underlie their antibacterial effect.

#### 3.2.3. In Vitro Toxicity Assessment

Materials for biomedical applications, based on many natural polysaccharides, in particular chitosan-based ones, are especially attractive because chitosan is a biocompatible, biodegradable polymer, which is characterized by the absence of immunogenicity and carcinogenicity, and chitosan is generally non-toxic [8]. However, the chemical modification of chitosan could potentially change the toxicity of chitosan: it would not be surprising if the conjugation of botulinum toxin to chitosan could result in a toxic chitosan derivative [56]. For this reason, it is believed that all new chitosan derivatives, especially those being advanced by the authors as medical polymers, must be subjected to at least a preliminary assessment of their *in vitro* toxicity. A very convenient method for the *in vitro* toxicity study is the 3-(4,5-dimethylthiazol-2-yl)-2,5-diphenyltetrazolium bromide (MTT) test, which allows for the estimation of the percentage of surviving cells after treatment with the sample under test [57].

In this work, HEK-293 cells for the MTT assessment of the toxicity of chitosan derivatives **TCs-0.10-L** and **TCs-0.30-L** were employed and compared with the polymers of the starting chitosan, with a viscosity average molecular weight of 15 kDa. Table 4 shows that the toxicity of HEK-293 cells for all test compounds increased with increasing sample concentrations. The lowest toxicity is characteristic of the starting chitosan: even at a concentration of 1000 μg/mL, 70 % of chitosan-treated cells survived. The introduction of the substituent into the chitosan backbone increased the toxicity of the synthesized polymers. An important proof of this fact is the increase in toxicity with the increasing degree of substitution. For example, at a test sample concentration of 300 μg/mL after treatment with chitosan, 92% of cells survive, with the low-substituted derivative **TCs-0.10-L**—88%, and with the moderately substituted derivative **TCs-0.30-L**—71%. The reduced toxicity of **TCs-0.10-L** when compared to **TCs-0.30-L**, was apparently due not only to the lower degree of substitution, but also to the state of **TCs-0.10-L** as self-assembled nanoparticles. In such nanoparticles, the hydrophobic substituent is usually located in the core, and the hydrophilic polymer backbone makes up the shell. This makes the substituents that provide the toxic effect less available for contact with a living cell.

In general, the data presented in Table 4 lead to the conclusion that the toxicity of leading polymer **TCs-0.10-L** (self-assembled into nanoparticles) differs slightly from the toxicity of the starting chitosan, which is usually considered a non-toxic biopolymer. Therefore, according to the results of the MTT test, **TCs-0.10-L** can be classified as a non-toxic polymer. In addition, it should be acknowledged that the non-toxicity of the polymer is demonstrated only at low concentrations.

#### 3.2.4. In Vivo Antibacterial Activity and Toxicity Studies

The abdominal peritonitis of bacterial infectious etiology is an extremely serious pathological process with a high probability of death; that is, it is essentially a complicated peritoneal sepsis [58]. On the other hand, model abdominal peritonitis is a very convenient test for the study of *in vivo* antibacterial activity [59]. After introducing a mixture of pathogenic microbes into the abdominal cavity of rats, the symptoms of acute peritonitis manifest quickly, which is very convenient for the conduct of the experiment. Literally, a few hours after the injection of the tested antibacterial compound into the abdominal cavity, the researcher observed a rapid disappearance of the symptoms (in the case of an effective antibacterial agent) or, conversely, a further increase in symptoms (when testing an ineffective sample) [60]. Thus, a few hours from the start of the treatment, it is possible to draw a conclusion about the effectiveness of the test compound and to predict, with the highest accuracy, the laboratory animal survival rate. The quantitative characteristic of this test is to count the number of colonies of the pathogenic bacteria, grown on agar after taking a sample of an abdominal exudate, 24 h after treatment. In the case of highly effective drugs, the colonies are either not observed or they are rare single units [59].

In the current work, Wistar rats with a microbial mixture of *E. coli* and *S. aureus* were infected. After infection, the rats showed all the symptoms expected in this case, including an increase in body temperature with a background increase in the respiratory rate and average contractions, as well as a swollen abdomen that was sharply painful on palpation. Thus, 24 h after infection, the rats were subjected to different intraperitoneal injection treatments: with equal concentrations of (*i*) a solution of 15 kDa of chitosan in its hydrochloride salt form, or (*ii*) polymer **TCs-0.10-L**, or (*iii*) a mixture of the commercial antibiotics, ampicillin and gentamicin (1:1). The experimental results are presented in Table 5.

The starting chitosan is characterized by the lowest *in vivo* antibacterial effect for the treatment of acute peritonitis: this polymer demonstrates a CFU per 1 mL of abdominal exudate value, which is only 1.6 times less than that for a blank experiment (i.e., the intraperitoneal infection of rats with a microbial mixture of *E. coli* and *S. aureus* without any subsequent treatment). The classical commercial antibiotics, i.e., ampicillin and gentamicin, have considerably greater effects. When infected rats were treated with antibiotics, only single colonies were observed. However, the **TCs-0.10-L** polymer turned out to be no less effective, and even more effective, as an antibacterial agent in the *in vivo* treatment of acute peritonitis than the commercial antibiotics. Thus, 24 h after the start of the treatment of rats with **TCs-0.10-L**, colonies that often grow after the plating of the abdominal exudate on agar were not detected. Apparently, the better antibacterial action of the **TCs-0.10-L** polymer can be explained by the slower removal of macromolecules and their nanoparticles from the body in comparison with the small molecules of the commercial ampicillin and gentamicin.

The *in vivo* toxicity of **TCs-0.10-L** in healthy, uninfected rats was assessed. It was observed that one daily intraperitoneal injection of the polymer (2000 mg/kg) for 14 days did not lead to any characteristic features of intoxication, did not change the respiratory or heart rate, and did not, in any way, affect food intake or general behavior in comparison with the rats of the blank experiment (injection of water instead of **TCs-0.10-L**). In addition, there were no significant changes observed in peripheral blood parameters in the treated rats when compared to the control group. Based on the experiments performed, it can safely conclude that the **TCs-0.10-L** is characterized by an LD50 > 2000 mg/kg, and this allows for its consideration as a low-toxic substance (hazard class IV).

## 4. Conclusions

The results of the current work can be considered from several perspectives.

Firstly, a fast and convenient route to tetrazole chitosan species, through the simple and efficient electrochemical coupling of hydrazone and an azide moiety, has been proposed. The simplicity of this synthetic operation and the high product yields make it attractive not only in chitosan chemistry but also in polymer chemistry in general. This advancement allows, over time, an optimism that this reaction will become an important and frequently used polymer-analogous transformation.

It is believed that there is no reason to fear or doubt the widespread adoption of electrochemical methods in polymer science. Given the current development of electrochemistry, such concerns are completely unjustified. Specifically, regarding the proposed reaction,

(1) The electrolyzer used is highly inexpensive and can even be constructed independently from readily available materials. (2) The electrodes are simple, traditional, and widely used, making them easily obtainable from commercial sources and reusable in a wide range of other electrochemical syntheses. (3) The proposed procedure for electrochemical synthesis is very straightforward and convenient, hence requiring no special training.

Secondly, a catalytic activity that is quite rare for chitosan; viz, tetrazole chitosan derivatives are highly effective catalysts for the aldol reaction. In the proposed catalytic procedure, water constitutes ~85% of the solvent, which certainly qualifies this catalysis as green chemistry. Additionally, chitosan or its derivatives are typically used as polymer supports for catalysts. However, in this work, a non-toxic chitosan derivative functions directly as a catalyst and notably as a metal-free catalyst. It is hoped that this example will inspire our colleagues to employ the use of chitosan and its derivatives not only as substrates but also as environmentally friendly organocatalysts, hence aligning with one of the key principles of green chemistry.

Thirdly, a pronounced antimicrobial activity in the electrochemically prepared, low-substituted tetrazole chitosan derivatives (**TCs-0.10-L**), which can self-assemble into nanoparticles, was ascertained. These nanoparticles exhibit a high positive zeta potential, thereby enabling them to severely damage bacterial membranes, leading to the leakage of cellular contents and subsequent bacterial death. In the *in vivo* experiments on rats, the high antibacterial activity of **TCs-0.10-L** was confirmed, along with their low *in vivo* toxicity. The tested polymers matched the antibacterial efficacy of the commercial antibiotics, i.e., ampicillin and gentamicin, thereby making them promising candidates for further preclinical studies. The polymers obtained are very attractive for the elaboration of antibacterial films for biomedical applications (tissue engineering scaffolds, wound dressings, burn dressings), especially after the modulation of their physicochemical properties under plasma.

Finally, the convenience and elegance of the electrochemical approach for chitosan chemistry have been practically demonstrated. This approach has been found to be promising for application in the chemistry of other natural or synthetic polymers, and this project is currently being pursued by this group.

## Figures and Tables

**Table 1:** Abbreviated names, degrees of substitution of the chitosan derivatives obtained, and molecular weights of the starting chitosan.

Hydrazone Chitosan Derivatives/Solubility	Tetrazole Chitosan Derivatives/Solubility	Molecular Weight of the Starting Chitosan, kDa	Degree of Substitution
**HCs-0.10-L**	**TCs-0.10-L**	15	0.10
**HCs-0.10-M**		60	0.10
**HCs-0.10-H**		160	0.10
**HCs-0.30-L**	**TCs-0.30-L**	15	0.30
**HCs-0.30-M**	**TCs-0.30-M**	60	0.30
**HCs-0.30-H**	**TCs-0.35-H**	160	0.30
**HCs-0.65-L**	**TCs-0.65-L**	15	0.60
**HCs-0.65-M**	**TCs-0.65-M**	60	0.60
**HCs-0.65-H**	**TCs-0.65-H**	160	0.60

**Table 2:** Catalytic activity of **TCs** species in the model reaction.

Tested Catalyst	Conversion, %				
	5 min	10 min	15 min	30 min	60 min
Chitosan	0	0	0	0	0
**TCs-0.10-L**	0	0	5	17	26
**TCs-0.30-L**	8	28	57	85	100
**TCs-0.30-M**	10	28	58	85	100
**TCs-0.35-H**	9	28	58	85	100
**TCs-0.65-L**	61	78	100		
**TCs-0.65-M**	58	80	100		
**TCs-0.65-H**	61	77	100		
**A**	70	85	100		

**Table 3:** Antibacterial effects of tetrazole chitosan derivatives.

Tested Sample	Inhibition zone, mm *	
	*Staphylococcus aureus*	*Escherichia coli*
Chitosan 15 kDa	12.7 ± 0.1	8.5 ± 0.1
Chitosan 60 kDa	10.3 ± 0.3	7.1 ± 0.1
Chitosan 160 kDa	10.2 ± 0.2	7.1 ± 0.2
**TCs-0.10-L**	27.8 ± 0.1	22.6 ± 0.3
**TCs-0.30-L**	14.6 ± 0.2	11.1 ± 0.2
**TCs-0.30-M**	13.2 ± 0.1	9.4 ± 0.3
**TCs-0.35-H**	13.2 ± 0.1	9.3 ± 0.1
**TCs-0.65-L**	13.5 ± 0.1	9.1 ± 0.2
**TCs-0.65-M**	13.7 ± 0.3	9.2 ± 0.2
**TCs-0.65-H**	13.7 ± 0.3	9.3 ± 0.2
**A**	14.4 ± 0.3	10.0 ± 0.3
Ampicillin	28.1 ± 0.3	-
Gentamicin	-	23.3 ± 0.3

* mean value ± SD, *n* = 3.

**Table 4:** Results of MTT assessment of *in vitro* toxicity.

Sample	Percentage of Surviving Cells at Various Concentrations of Test Sample *		
	10 µg/mL	300 µg/mL	1000 µg/mL
Chitosan	100	92	70
**TCs-0.10-L**	95	88	66
**TCs-0.30-L**	89	71	48

* the relative standard deviation for all points is 5–10%.

**Table 5:** *In vivo* acute peritonitis test.

Sample	CFU per mL of Exudate (7 h after Treatment) *
Blank experiment	2860
Chitosan	1780
**TCs-0.10-L**	0
Ampicillin and gentamicin (1:1)	30

* the relative standard deviation for all points is 5–10%.

## Data Availability

The original contributions presented in this study are included in the article. Further inquiries can be directed to the corresponding authors.
